# Biological Accessibility and Analytical Recovery in Myofibrillar Protein Loss During Skeletal Muscle Atrophy

**DOI:** 10.3390/biom16101398

**Published:** 2026-09-25

**Authors:** Thanh Huu Phan Ngo, Wan Lee

**Affiliations:** 1 Department of Biochemistry, Dongguk University College of Medicine, 123 Dongdae-ro, Gyeongju 38066, Republic of Korea; thngo139@gmail.com; 2 Section of Molecular and Cellular Medicine, Medical Institute of Dongguk University, Dongguk University College of Medicine, 123 Dongdae-ro, Gyeongju 38066, Republic of Korea

**Keywords:** skeletal muscle, atrophy, thin filament, actin dynamics, ubiquitin–proteasome system, myofibril extraction

## Abstract

Myofibrillar loss during skeletal muscle atrophy is often described as selective. MuRF1 is considered to remove thick filament proteins preferentially, whereas thin filament proteins are assigned to other ligases or appear relatively spared. We reassess whether this pattern demonstrates molecular specificity or is partly explained by the state in which a protein is encountered and measured. Several observations motivate this analysis. Actin can be polyubiquitinated by MuRF1 even though loss of assembled thin filament proteins is not MuRF1-dependent in several models. Apparent preferential myosin loss varies with extraction chemistry, normalization, and the combination of catabolic stimuli with inactivity. KLHL40 and KLHL41 preserve major thin filament proteins, although the direct evidence comes mainly from development and myopathy. Turnover and ubiquitin-remnant proteomics quantify molecules recovered from defined fractions and do not, on their own, distinguish degradation from redistribution. We therefore distinguish two biological determinants from one observer-side analytical layer. Assembly-dependent exposure and stability or damage-state regulation influence biological availability. Extraction, fractionation, detectability, and normalization determine analytical recovery. Ubiquitination remains distinct from degradation because chain architecture, deubiquitination, extraction, unfolding, and productive disposal can still determine fate. This framework retains ligase specificity while defining the controls needed to establish preferential degradation in intact muscle.

## 1. Introduction

Skeletal muscle atrophy reduces force-generating capacity by removing a large protein mass while leaving recognizable sarcomeric order, at least during early and moderate wasting. Genetic and transcriptional studies established MuRF1 and atrogin-1/MAFbx as central atrogenes [1,2,3]. FoxO signaling coordinates proteasomal and autophagic programs [4,5]. Autophagy also has a homeostatic role. Its loss damages sarcomeres and can worsen muscle loss during denervation or fasting [6]. Reviews of the ubiquitin–proteasome system and its E3 ligases define the major degradative framework [7,8,9]. Calcium-dependent proteases, caspases, and chaperone systems provide additional routes [10,11,12]. Ordered sarcomeres can persist during early wasting. This raises a central question. Which step limits access to a structural protein before its final route of disposal is assigned?

The most influential experimental formulation of this asymmetry came from Cohen and colleagues, who showed that MuRF1-dependent ubiquitylation drives an ordered loss of thick filament proteins during denervation and fasting, while actin, tropomyosin, troponins, and alpha-actinin declined similarly in wild-type and ligase-defective muscle [13]. In later summaries, this result is often compressed into the statement that MuRF1 recognizes thick, but not thin, filament substrates. The compression is understandable, but it removes a distinction made in the original study. Purified or released proteins can be competent substrates while the same molecules are protected when incorporated into a highly ordered myofibril. The measured phenotype reflects at least two variables. They are molecular recognition and physical access.

Several observations make this distinction consequential. Polge and colleagues reported MuRF1-dependent polyubiquitination and degradation of muscle actin in cellular, biochemical, and biopsy-associated experiments [14]. Low ionic strength extraction can also generate or magnify apparent preferential myosin depletion by leaving thick filaments in insoluble material [15]. A third line of evidence comes from BTB-BACK-kelch proteins. KLHL40 and KLHL41 can preserve major thin filament proteins [16,17,18]. Studies of KBTBD13 and CUL3 connect related adaptors to sarcomeric function [19,20,21], while genetic and clinical work extends this association to nemaline myopathy [22,23,24,25]. These observations do not invalidate the founding genetic work. They show that absence of net loss in an assay cannot be equated with absence of substrate recognition.

This review does not attempt another catalog of myofibrillar proteolysis. Aweida and Cohen have already provided a detailed stepwise account of how the UPS dismantles filamentous myofibrils, including desmin depolymerization, ubiquitination, extraction by AAA-ATPases, and proteasomal degradation [26]. Our distinct question is why a component is not reached, or is not observed to be reached, in the first place. We focus on skeletal muscle and use cardiac, developmental, myopathy, or non-muscle studies only when they clarify a potentially conserved quality control principle. Such evidence is identified as mechanistic support and not as direct evidence for adult skeletal muscle atrophy. Fiber-type-specific atrophy responses are not reviewed systematically. Fiber-type-associated structural differences are considered only when they illustrate how the same protein can occupy different sarcomeric environments.

## 2. MuRF1-Dependent Thick Filament Loss and Actin Ubiquitination

### 2.1. Ordered Thick Filament Loss Is Already an Accessibility Model

Cohen et al. generated mice expressing a RING-deleted MuRF1 that retained binding capacity but lacked effective ubiquitin-ligase activity [13]. Following denervation or fasting, wild-type muscle lost myosin-binding protein C (MyBP-C) and myosin light chains before major loss of myosin heavy chain (MyHC). The corresponding losses were blunted when MuRF1 function was disrupted. Thin filament and Z-disk proteins, by contrast, declined without the same dependence on MuRF1. These findings established both a ligase-dependent component of atrophy and an ordered sequence within the thick filament.

The sequence matters as much as the substrate list. MyHC in an intact filament is not equivalent to soluble MyHC. Associated proteins, lattice packing, and the supramolecular environment can conceal lysines, degrons, or binding surfaces. Removing MyBP-C and light chains can expose MyHC to subsequent ubiquitination and extraction. Thus, even within the thick filament, the first event is not unrestricted ligase recognition of every component. A structural transition precedes efficient degradation. Commentary and later mechanistic work have accordingly described myofibril destruction as loss of stabilizing pieces without immediate collapse of the entire lattice [26,27,28].

This principle is supported by older biochemical observations that actin and myosin are rapidly degraded when purified but resist ubiquitin-dependent proteolysis in actomyosin complexes or myofibrils [13]. It is also compatible with proteomic mapping. In denervated muscle, ubiquitin-remnant enrichment identified a strong signal in sarcomeric proteins, including MyHCs, myomesins, and titin [29]. Titin carried an early concentration of upregulated diglycine sites near its C-terminus, which anchors at the M-band, creating a nonuniform spatial gradient across the protein [29]. Position within the assembly can influence where degradation begins.

MuRF1 also belongs to a partially overlapping ligase family. MuRF1/MuRF2 double deletion produces marked cardiac and milder skeletal hypertrophy with altered metabolic regulation [30]. Combined loss of MuRF1 and MuRF3 causes myosin accumulation and striated-muscle myopathy [31], whereas MuRF2/MuRF3 double deficiency disrupts myofibrillar structure and reduces skeletal-muscle force [32]. MuRF3 also regulates microtubule stability during myogenic differentiation [33]. These genetic data support functional overlap but do not establish that every shared effect requires MuRF heterodimerization. Nuclear, metabolic, and microtubule-related functions are noted here because they complicate single-ligase inference. A comprehensive treatment of these non-myofibrillar roles lies outside the present focus on filament turnover.

### 2.2. Actin Is a Competent MuRF1 Substrate Under Other Conditions

Polge et al. directly challenged the idea that muscle actin lies outside the MuRF1 substrate repertoire [14]. In dexamethasone-treated myotubes, actin accumulated polyubiquitin conjugates, MuRF1 depletion stabilized epitope-tagged actin, and recombinant MuRF1 ubiquitinated actin in vitro. The study also detected ubiquitinated actin in human skeletal muscle material. These results demonstrate ubiquitination competence, but the experimental contexts included tagged actin, soluble or myofibrillar-enriched lysates, dexamethasone-treated cells, and recombinant reactions. They do not address the same structural and temporal questions as the in vivo knock-in experiments.

The two reports are best treated as measurements of different conditional probabilities. Cohen et al. asked whether loss of proteins incorporated into native myofibrils during defined atrophy models requires MuRF1 [13]. Polge et al. asked whether actin can bind, become polyubiquitinated by, and undergo degradation through a MuRF1-dependent route under biochemical and cellular conditions [14]. A protein may answer yes to the second question and no to the first if assembly suppresses access. Conversely, a loss-of-function phenotype in vivo can persist even when MuRF1 can biochemically modify the isolated protein.

This distinction also prevents an overly sharp division between thick and thin filament ligases. TRIM32 has been implicated in removal of desmin, actin, tropomyosin, troponins, and alpha-actinin, with desmin depolymerization facilitating later myofibrillar breakdown [26,27,28,34]. MuRF1 ubiquitylome studies likewise identify a broader set of candidate proteins than the small group whose steady-state loss has been genetically demonstrated [35]. Interactomes, in vitro ubiquitination, ubiquitin-remnant enrichment, and genotype-dependent abundance each supply different evidence. They should not be collapsed into a single label of “substrate.”

### 2.3. Interpretation of the Divergent Findings

Three conclusions remain secure. First, MuRF1 is required for the efficient loss of specific thick filament components in several atrophy settings [13,36]. Second, actin can be polyubiquitinated in a MuRF1-dependent manner under particular cellular and biochemical conditions [14]. Third, incorporation into an ordered myofibril changes the degradability of contractile proteins [26,27,28]. The unresolved variable is access. Access depends on the structural state in which the ligase encounters the protein, the molecular partners that cover it, and the extraction procedure that makes it visible to the investigator.

Accordingly, thin filament sparing should be described operationally. It means that a measured pool declined less, or declined independently of the tested ligase, under a specified model and extraction method. It does not, by itself, prove that the ligase lacks affinity for every biochemical form of that protein. This more limited wording preserves the genetic evidence while avoiding a specificity claim that the actin data do not support.

### 2.4. Dismantling Requires Release, Extraction, and Disposal

Recognition by an E3 ligase is only one event in myofibril loss. The proteasome accepts unfolded polypeptides through a narrow axial channel and cannot ingest an intact actomyosin lattice. Pharmacological inhibition shows that proteasome activity accounts for a large share of accelerated proteolysis in atrophying muscle, but it identifies the terminal disposal system and does not identify the step that makes a sarcomeric protein available to it [26,37]. A mechanistic model must separate structural loosening, covalent marking, extraction from the assembly, unfolding, and final destruction. These events may be ordered differently for different substrates and models.

Caspase-3 provides one proposed route into this sequence. In catabolic models, limited actomyosin cleavage generated a characteristic approximately 14 kDa actin fragment and increased subsequent proteasome-dependent degradation [38]. Caspase-3 deficiency also attenuated denervation atrophy in vivo [11]. These findings do not mean that caspase-3 universally initiates myofibril breakdown. Calpains, desmin remodeling, and other release mechanisms differ among models. They do show why a full-length actin band, a cleavage fragment, and loss of total actin are distinct readouts. Cleavage may increase access without yet demonstrating either ubiquitination or complete degradation.

Calcium-dependent myofilament release supplies a complementary example. Septic muscle showed Z-band disruption and release of actin- and myosin-containing material, while interventions that limited calcium-dependent proteolysis reduced the response [10,39]. Importantly, the calpain-inhibitor phenotype occurred without proportional suppression of atrogin-1/MAFbx or MuRF1 expression [40]. Expression of an atrogene and physical availability of its substrates can be regulated on different axes. A strongly induced ligase may have little effect on a filament that has not been released, whereas modest ligase abundance may suffice once an assembly barrier is removed.

Extraction from a marked assembly is itself an active step. Dominant-negative p97/VCP reduced denervation- and fasting-induced atrophy and trapped ubiquitinated thin and thick filament components together with p97 cofactors, supporting a segregase function at the myofibril [41]. The delayed denervation program described by Volodin et al. similarly required desmin depolymerization, PAX4-dependent induction, and p97/VCP [28]. METTL21C-dependent trimethylation of p97 altered its assembly and ATPase activity and modified protein quality control phenotypes in skeletal muscle [42]. These studies establish extraction capacity as a biological variable and not a technical afterthought.

Accessibility therefore remains one regulatory layer and should not be assumed to be the sole rate-limiting step. After a protein is exposed and ubiquitinated, chain architecture can recruit different effectors, deubiquitinating enzymes can edit or remove the signal, and p97/VCP-mediated extraction may still be required before unfolding and productive proteasome engagement [26,41]. Damaged complexes can also enter chaperone-assisted autophagy instead of the proteasome. The fate of an accessible substrate must therefore be tested directly and cannot be inferred from ubiquitination alone.

## 3. Model and Method Dependence of Apparent Selectivity

### 3.1. Ionic Strength Can Create Apparent Preferential Myosin Loss

The strongest warning comes from a basic measurement issue. Myosin is poorly extracted from intact thick filaments by low ionic strength buffers and becomes more recoverable as ionic strength rises. Cosper and Leinwand compared conventional low-salt lysis with a high-salt myosin extraction buffer and showed that low-salt supernatants substantially underestimate MyHC [15]. Studies reporting extreme preferential MyHC depletion often quantified only these supernatants.

The underlying accounting is important. Myosin constitutes roughly 30% of total muscle protein, but a band normalized to equal lysate protein reports concentration within the recovered material, not total MyHC content per muscle. A 50% fall in muscle mass can produce a 50% fall in total MyHC content even if the MyHC-to-total-protein ratio is unchanged. Conversely, an approximately 80% fall in a normalized MyHC band implies additional disproportionate depletion and is highly sensitive to protein-specific recovery. With adequate high-salt extraction, Cosper and Leinwand did not observe selective MyHC depletion in the cachexia model studied [15]. The result does not show that preferential myosin loss never occurs. It shows that extraction and denominator must be treated as part of the biological inference.

This issue matters because Western blots are often normalized to soluble material recovered after lysis. If the fraction of MyHC entering that material changes with filament packing, post-translational modification, or buffer composition, the normalized band reports both extractability and abundance. Total-protein staining cannot repair a protein-specific recovery failure. Pellet analysis, high-salt extraction, or quantitative recovery controls are required.

Titin is especially vulnerable to incomplete analytical recovery. Its multimegadalton size makes extraction sensitive to solubilization, mechanical shearing, gel composition, electrophoretic entry, and transfer efficiency. Regional mass-spectrometric coverage also depends on digestion and peptide detectability. Dedicated titin electrophoresis and paired assessment of intact protein, fragments, and regional peptides are therefore needed for quantitative inference [43]. A stable titin band or a regional ubiquitin-remnant map alone cannot establish uniform whole-molecule abundance or accessibility.

### 3.2. Preferential Loss Depends on the Combination of Insults

Model dependence provides a second qualification. Yamada et al. found that cancer cachexia alone did not produce preferential myosin loss under their analytical conditions, and dexamethasone alone likewise did not reproduce the phenotype. Preferential loss appeared when the systemic catabolic stimulus was combined with denervation [44]. Electrical stimulation can attenuate preferential myosin loss in steroid-denervation models, further linking the phenotype to inactivity and neuromuscular disconnection and arguing against a universal cachectic program [45].

Clinical and extreme-physiology observations also resist a single rule. Acute quadriplegic myopathy is often characterized as an acquired myosinopathy, and intensive-care interventions can produce marked motor-protein dysfunction [46,47]. Spaceflight alters thick and thin filament isoforms in a muscle- and fiber-type-dependent manner [48]. Recent denervation work identified RBP4-STRA6-JAK2-STAT3 signaling as one contributor to muscle loss [49]. This pathway does not establish selective myosin degradation, but it reinforces that denervation changes the signaling environment in which systemic catabolic stimuli act. These settings differ in neural drive, mechanical load, glucocorticoid exposure, inflammation, nutrition, and time. A substrate-selectivity statement detached from the model discards variables that may determine whether the myofibril becomes accessible.

The practical conclusion is modest but important. Preferential myosin loss is a model- and method-dependent phenotype, not a constitutive property of muscle atrophy. It should be reported together with the catabolic stimulus, neural and loading state, duration, muscle, extraction buffer, analyzed fraction, and denominator. Table 1 compares the principal models discussed here and makes those boundaries explicit.

### 3.3. Fraction Redistribution Can Mimic Synthesis or Negative Breakdown

Dynamic proteome profiling adds a more subtle example. Camera and Burniston combined protein-specific synthesis with changes in abundance to estimate breakdown after a high-fat diet and resistance exercise [50]. Several proteins generated mathematically negative breakdown estimates. Negative degradation is not biologically meaningful. The authors interpreted these values as evidence that proteins or premyofibrillar complexes had moved into the analyzed myofibrillar fraction. Alpha-actin and troponin C2 were among proteins for which assembly or redistribution could explain an excess increase in the myofibrillar pool [50].

This phenomenon reveals that fractionation is not a neutral window onto turnover. A soluble regulatory protein can appear to be lost when it enters the filament, whereas an incorporated protein can appear stable when a newly assembled pool replaces degraded molecules. Similarly, a component released from damaged myofibrils may disappear from the myofibrillar fraction before it is degraded. Without paired analysis of soluble, insoluble, and total pools, compartment transfer is mathematically indistinguishable from synthesis or breakdown in a single fraction.

Table 2 summarizes the interpretive boundaries of commonly used measurements. The point is not that the cited assays are unreliable. Each assay answers a legitimate question, but the answer is narrower than the biological label often attached to it.

### 3.4. Fraction Completeness and Mass Balance

The denominator creates a second accounting problem. Equal-protein loading answers how much target is present per unit of recovered protein. It does not answer how much target remains per muscle, per fiber, or per original tissue mass. A smaller atrophic muscle can retain the same target-to-total-protein ratio while losing a large absolute amount. Conversely, poor extraction of a single highly abundant filament can disproportionately lower its band. Matched high- and low-salt extraction, analysis of both supernatant and pellet, and normalization back to the starting tissue are necessary when preferential loss is the claim [15,37].

Ubiquitin-remnant proteomics requires a parallel denominator. A larger diglycine-peptide signal may reflect higher site occupancy, greater abundance of the parent protein, improved peptide recovery, or movement of the modified species into the analyzed fraction. Quantifying the corresponding unmodified peptide or total protein in every fraction does not solve all chain-topology and E3-attribution questions, but it distinguishes modification stoichiometry from simple abundance change [29,35]. Spike-in recovery standards and orthogonal immunoblotting of pellets can further reveal protein-specific extraction failures.

## 4. Structural Stabilization and Damage-State Regulation

### 4.1. KLHL40 Stabilizes Key Thin Filament Proteins

BTB-BACK-kelch proteins commonly act as substrate adaptors for CUL3-RING ubiquitin ligases. KBTBD13 and related adaptors connect this family to sarcomeric mechanics and CUL3 activity [19,20,21]. Other genetic and clinical studies link kelch proteins to myofibrillar preservation and nemaline myopathy [22,23,24,25]. KLHL40 localizes to sarcomeric regions, binds nebulin (NEB) and leiomodin-3 (LMOD3), and increases their protein abundance without corresponding mRNA changes [17]. KLHL40-deficient mice and patients with severe KLHL40 mutations show marked reductions in NEB and LMOD3, muscle weakness, and sarcomeric abnormalities [17].

The mechanism differs for each target. KLHL40 reduces K48-linked polyubiquitination of LMOD3 and protects it from proteasome-mediated degradation [17]. NEB stabilization appears less directly linked to proteasome inhibition and may involve proper folding or the prevention of aggregation. Thus, the safe conclusion is not that KLHL40 blocks ubiquitination of all thin filament proteins. Instead, KLHL40 appears to promote a more stable state for NEB and LMOD3, with direct evidence of reduced LMOD3 ubiquitination and a distinct, less resolved mechanism for NEB.

This directly regulates structural stability but does not show that KLHL40 physically masks a degradative binding site. By promoting folding, assembly, or partner retention, KLHL40 can indirectly reduce the exposure of NEB or LMOD3 to quality control machinery. The distinction matters because stabilization can influence accessibility without being synonymous with it.

### 4.2. KLHL41 Separates Adaptor Ubiquitination from Substrate Ubiquitination

KLHL41 provides a second, mechanistically different case. It binds nebulin repeats and stabilizes a nebulin fragment that otherwise has a short half-life of approximately 6 h in cycloheximide-chase experiments [16]. KLHL41 itself undergoes K48-linked polyubiquitination on its BTB domain, and this modification is required for efficient nebulin stabilization. The nebulin substrate is not thereby marked for degradation. The term “nonproteolytic ubiquitination” in the original title refers to a ubiquitination-dependent stabilizing mechanism centered on KLHL41, not to nonproteolytic ubiquitination of nebulin.

The data favor a chaperone-like mechanism in which KLHL41 prevents nebulin aggregation and supports sarcomere integrity [16]. Nevertheless, the mechanistic boundary should remain explicit. Peer review of the study noted that an indirect model, in which KLHL41 degrades a third factor that would otherwise destabilize nebulin, was not completely ruled out. Proteasome or autophagy inhibition did not rescue the nebulin fragment in the absence of KLHL41, arguing against a simple version of that model, but not proving that every indirect route is absent [16]. The appropriate claim is that KLHL41-dependent autoubiquitination is required for nebulin stabilization and is consistent with chaperone-like protection.

### 4.3. The Same Adaptor Can Protect One Partner and Destroy Another

KLHL41 is not simply an anti-degradation protein. It also regulates nebulin-related anchoring protein (NRAP), a developmental chaperone of myofibril assembly. KLHL41 promotes NRAP ubiquitination and degradation. When KLHL41 is deficient, NRAP persists, disrupts the KLHL40-nebulin interaction, and worsens nemaline-myopathy pathology [18]. Reducing NRAP improves aspects of the disease phenotype [18]. The same molecular environment uses controlled degradation to end one chaperone program while preserving a mature structural protein.

This bidirectionality undermines a binary classification of proteins as degradative E3 adaptors or stabilizing chaperones. The functional outcome depends on the partner, developmental time, complex composition, and assembly state. KBTBD13 extends the overlap. Recent genetic and therapeutic work links KBTBD13 dysfunction to abnormal sarcomere organization and impaired muscle-relaxation kinetics, while biochemical studies also place it in a CUL3-containing ubiquitin-ligase complex [20,21]. Cullin-3 activity is broadly required for striated-muscle function, and mutations in several kelch proteins cause myofibrillar disruption [19,22,23,24,25]. The boundary between structural constituent, access regulator, and ubiquitin-system component is permeable.

### 4.4. Chaperone Systems Define Substrates by Damage State

CASA supplies a parallel principle. BAG3-containing complexes recognize mechanically damaged or unfolded proteins, such as filamin C, and coordinate chaperone-assisted handling, ubiquitination, autophagic delivery, and replacement [51]. In cardiomyocytes, reduced BAG3-mediated sarcomeric turnover impairs contractility, illustrating that protection cannot mean indefinite retention. Damaged components must be made accessible at the appropriate time [52]. Titin quality control similarly combines chaperones, ubiquitin pathways, autophagy, and mechanically exposed domains [53]. Ozz-dependent regulation of embryonic myosin replacement further demonstrates that E3 ligases can control exchange rates within myofibrils rather than simply bulk destruction [54].

These examples suggest that protein state contributes to specificity in addition to sequence identity. A folded, force-bearing, properly incorporated molecule can be protected, while the same sequence may become a substrate after unfolding, release, oxidation, cleavage, or loss of chaperone support. For the thin filament, an apparently spared pool may therefore reflect successful quality control rather than absence of degradative capacity.

The main limitation is equally important. Direct evidence for KLHL40 and KLHL41 comes mainly from developmental and nemaline myopathy systems [16,17,18]. Related genetic studies support a broader role for kelch proteins in sarcomeric biology [19,20,21]. Clinical, genetic, and review evidence concerning kelch proteins [22,23,24,25] and thin filament disease [55,56,57] provides additional context, but none establishes an adult atrophy pathway. Extension to denervation, cachexia, or aging remains a hypothesis. Dedicated protection clearly exists in muscle. The available evidence does not show that KLHL proteins explain thin filament sparing in every wasting condition.

### 4.5. Oxidation and Force-Dependent Damage Can Change Structural State

A substrate can change degradability without changing its amino-acid sequence or binding partner. MICAL-family monooxygenases provide a clear biochemical example. Mical binds F-actin and promotes disassembly, and direct oxidation of conserved actin Met44 severs filaments and limits repolymerization [58,59]. The modification is not inevitably terminal. SelR/MsrB can reduce Mical-oxidized actin and restore polymerization [60]. Oxidation therefore creates a reversible structural transition that could alter exposure to quality control machinery.

The transition is amplified by actin-disassembly proteins. Mical-mediated oxidation increases cofilin binding and severing, while MICAL1 oxidation can overcome protection by tropomyosin and permit rapid severing [61,62]. Single-filament measurements also show that ADF/cofilin promotes depolymerization from both filament ends and alters capping-protein behavior [63]. These biochemical and non-muscle cellular studies establish coupling among oxidation, side-binding proteins, and end regulation. They do not demonstrate that MICAL-driven oxidation initiates adult skeletal muscle atrophy or that depolymerized actin is necessarily ubiquitinated and degraded.

Skeletal muscle-cell evidence now links MICAL1 to actin-state control during C2C12 differentiation. MICAL1 depletion increased F-actin, altered YAP1 localization, prolonged proliferation, and impaired myogenic differentiation [64]. This finding supports a role for MICAL1 in muscle-cell actin remodeling, but it does not test adult atrophy, persistent Met44 oxidation, or the ubiquitination susceptibility of depolymerized actin. We did not identify direct evidence that MICAL-dependent oxidation remains persistently elevated during skeletal muscle atrophy or commits actin to proteasomal degradation. Redox modulation is therefore a testable candidate rather than an established therapeutic node.

Mechanical damage produces an analogous state definition. Tension can unfold cytoskeletal proteins and recruit BAG3-centered CASA for ubiquitin-dependent autophagic sorting [65]. BAG3 and Hsc70 also stabilize the barbed-end capping protein CapZ under mechanical stress. BAG3 dysfunction increased CapZ degradation and myofibrillar vulnerability in a cardiac model [66]. These findings suggest that quality control may recognize damaged conformations or destabilized complexes instead of a constitutively exposed sequence. A force-bearing filament can be shielded at baseline and become accessible after oxidation, uncapping, unfolding, or partner loss.

## 5. Measurements of Myofibrillar Turnover and Recovery

### 5.1. Protein-Specific Turnover Provides a Baseline, Not a Direct Degradation Readout

Stable-isotope labeling and mass spectrometry have advanced muscle biology beyond the limitations of mixed-protein synthesis rates. In a 2025 BMC Methods study, Stansfield et al. quantified fractional turnover rates from 3944 peptides representing 935 proteins in vivo in three physically active young men [67]. The median fractional turnover rate was 4.3% per day, with first- and third-quartile rates of 2.52% and 7.84% per day, respectively. The study further argued that mole and absolute turnover rates can change the biological interpretation because fractional values do not account for protein abundance [67].

The dataset is valuable but should not be overextended. It is a small physiological-methods study in healthy participants, not a measurement of atrophic muscle. Fractional turnover describes flux through a pool. It does not identify the protease, distinguish degradation from inter-pool transfer, or reveal whether a low value reflects molecular shielding, slow synthesis, limited peptide recovery, or averaging across proteoforms [67,68]. Protein-specific estimates are also sensitive to the definition of the pool and to changes in abundance.

Exercise studies provide complementary magnitudes. Camera and Burniston reported average myofibrillar synthesis values around 1.09% per day without resistance exercise and 1.58% per day with resistance exercise in their experimental design [50]. Other studies place mixed or myofibrillar synthesis in a similar approximate range, but synthesis cannot be used as a synonym for turnover when abundance is changing [50,67,69]. Dynamic SILAC studies in non-muscle cells demonstrate the broader feasibility of proteome-wide half-life estimation while also showing that cell-culture values cannot be imported uncritically into adult muscle [68]. Developmental imaging of Z-band proteins and work on sarcomeric actin dynamics further show that incorporation and exchange are protein- and location-specific [70,71].

### 5.2. Denervation Ubiquitinomics Maps Modification, Not Fate

Lang et al. examined early denervation and observed approximately 20% gastrocnemius mass loss by day 7 [29]. Lysine-epsilon-diglycine enrichment mapped 2328 sites on 667 proteins, including extensive sarcomeric modification. Titin carried 418 mapped remnants, with early regulated sites concentrated toward its M-band-associated C-terminus [29]. This distribution is consistent with spatial heterogeneity of modification within the sarcomere, but it does not by itself establish spatially ordered accessibility. Protein abundance, proteolytic digestion, peptide recovery, ionization, and regional sequence coverage can also shape the observed gradient.

#### Titin as a Structural and Analytical Special Case

The M-band-associated C-terminus of titin is embedded in a dense signaling and anchoring environment. A regional increase in ubiquitin-remnant sites could reflect locally exposed domains, damage, binding-partner changes, or preferential recovery of informative peptides. Titin is also unusually difficult to quantify because intact molecules and large fragments require specialized extraction and electrophoresis, while routine blots and mass spectrometry sample only a fraction of the sequence [43]. The Lang et al. pattern is therefore a useful spatial hypothesis that should be tested with regional total-protein normalization, chain characterization, intact-titin analysis, and matched soluble and insoluble fractions [29].

Yet a diglycine remnant does not specify the chain topology, the responsible E3, proteasomal commitment, or the degradation rate. Highly abundant proteins generate more detectable peptides, and missing sites may reflect digestion, ionization, peptide length, or database assignment. Comparing ubiquitination with total abundance and assembly state is essential. MuRF1 overexpression-based ubiquitylome mapping broadens the candidate substrate set, but likewise cannot on its own prove direct ligase-substrate pairing or destruction within an intact myofibril [35].

### 5.3. Turnover of Structural-State Regulators Remains Unmeasured

Thin filament stability depends on polymerization, severing, capping, monomer handling, anchoring, and repair. Cofilin-2 is required for normal sarcomere organization and muscle maintenance. CFL2 mutations cause nemaline myopathy, and loss models produce progressive actin disorganization [72,73,74,75]. Non-muscle cofilin-1 also contributes to sarcomeric integrity under specific disease conditions [76]. Twinfilin can drive actin-barbed-end depolymerization, COTL1 antagonizes cofilin in actin-network remodeling, and drebrin has a demonstrated role in myotube formation [77,78,79]. Reviews of thin filament assembly and length control explain how related regulators influence filament state and disease [55,56,57].

The proteins discussed in this section are representative and do not constitute an exhaustive set. CFL2, twinfilin, COTL1, and drebrin were selected because they cover filament severing, barbed-end depolymerization, cofilin regulation, and filament organization. Capping proteins are included as an additional class because end occupancy can regulate subunit exchange. Profilin, tropomodulins, leiomodins, and nebulin are discussed separately as monomer-handling, end-regulating, or structural-stabilizing proteins. Placement in this framework does not imply that any of these proteins is already established as a degradation regulator during adult muscle atrophy.

What remains poorly characterized is their degradation in atrophy. For this narrative review, targeted PubMed searches through 15 September 2026 were complemented by reference-list screening of relevant primary studies and reviews. Searches combined each protein name and alias with skeletal muscle, atrophy, half-life, turnover, degradation, ubiquitin, proteasome, and autophagy. We did not identify a study that directly measured an atrophy-dependent half-life and degradation route for CFL2, twinfilin, COTL1, or drebrin in skeletal muscle. This bounded search does not prove that no relevant experiment exists. The closest quantitative thin filament-associated observation in the reviewed literature is the fraction-redistribution problem described for profilin-1, actin, and troponin C2 after resistance exercise [50]. That study did not examine atrophy and does not yield an interpretable degradation rate.

The gap is mechanistically important because these proteins can determine the assembly state that controls access. If an actin-severing or monomer-handling protein turns over rapidly during denervation, the resulting filament-state transition could precede visible actin loss. Conversely, long-lived capping or stabilizing proteins could preserve filament architecture despite strong induction of ubiquitin ligases. Actin and tubulin abundance are themselves subject to multilayered autoregulation, including transcriptional and post-transcriptional feedback [80]. Such homeostatic control further decouples steady-state abundance from degradation rate.

### 5.4. End Regulators Define Thin Filament Structural State

The ends of the sarcomeric actin filament are small molecular territories that disproportionately control stability. CapZ occupies the barbed end at the Z-line, and inhibition of CapZ-actin binding delays or disrupts patterned thin filament assembly without equivalently blocking thick filament organization [81,82]. Tropomodulin occupies the pointed end, where its interaction with actin, tropomyosin, and the N-terminus of nebulin limits subunit exchange [83,84]. These proteins are not simply static labels for filament ends. They define whether addition, loss, and access by disassembly factors are kinetically permitted.

Thin filament length is also not a universal constant. Human biopsies show muscle- and fiber-type-associated variation, including nebulin-free pointed-end extensions [85]. Nebulin-deficient mouse muscles have shorter filaments, impaired force, and structural abnormalities, and related patient and mouse models connect reduced nebulin abundance or altered nebulin structure to nonuniform length and weakness [86,87,88,89]. These observations establish that the same actin sequence can reside in distinct structural neighborhoods depending on fiber type, nebulin coverage, and disease state.

Pointed-end control is buffered by isoform compensation and competition. Tmod1 and a leiomodin isoform switch can compensate Tmod4 deletion, whereas depletion of Tmod1 elongates skeletal-muscle thin filaments [90]. Leiomodin has strong actin-nucleating activity, LMOD3 loss shortens and disorganizes skeletal thin filaments, and structural work supports a ‘leaky cap’ that competes with tropomodulin and permits limited pointed-end elongation [91,92,93,94]. The functional state of the pointed end depends on relative occupancy and complex composition, not merely on the presence of actin.

Nebulin also alters the filament’s physical state along its length. Conditional nebulin loss reduces thin filament stiffness and perturbs tropomyosin-troponin movement and cross-bridge recruitment [95]. A disease-linked substitution in the shared leucine-rich-repeat architecture of tropomodulin and leiomodin disrupts actin regulation and abolishes the expected length control functions [96]. Together, these results define multiple ways covering, capping, stiffness, and end geometry can alter actin surface exposure without changing E3-ligase identity.

Structural stability and accessibility are related but not interchangeable. End-binding proteins and nebulin directly influence the probability of depolymerization, elongation, or mechanical failure. They affect accessibility only indirectly when those structural changes expose binding surfaces, release subunits, or alter encounters with degradation machinery. The cited studies establish structural regulation more directly than they establish proteolytic access. CapZ, tropomodulin, leiomodin, and nebulin should therefore be considered candidate upstream determinants of accessibility rather than proven degradation gates.

### 5.5. Recent Evidence Reinforces Model and State Dependence

Several studies published since 2021 refine the central framework without overturning it. In human muscle, beta2-adrenergic stimulation and resistance training produced overlapping proteome remodeling, with KLHL40 and KLHL41 among the most strongly increased proteins, and KLHL41 knockdown attenuating beta2-agonist-induced myotube hypertrophy [97]. Cryo-electron tomography of intact myofibrils resolved nebulin along the thin filament and provided direct structural support for its stabilizing role [98]. Genetic reduction in NRAP improved sarcomeric organization and function in nebulin-deficient zebrafish [99]. Skeletal-muscle loss of Lmod2 shortened soleus thin filaments and reduced force, with compensatory Lmod3 upregulation [100]. Human redox proteomics further associated cysteine oxidation at nebulin, myomesin, alpha-actinin, and actin sites with measures of mobility and muscle function in older adults [101]. These data strengthen the links among structural state, stress, and muscle function, but they do not establish protein-specific degradation routes during adult atrophy.

Recent atrophy studies also demonstrate that model labels conceal distinct upstream programs. Cancer cachexia can engage OSM-OSMR-EDA2R-NIK signaling [102]. Sepsis can engage IL-6-gp130-JAK2-STAT3 signaling and alter MuRF1 protein abundance without an equivalent change in Trim63 transcript [103]. Fasting involves CARM1-dependent regulation of autophagy and mitophagy [104]. A recent synthesis indicates that, during dexamethasone-induced atrophy, PDK4 signaling can promote myogenin phosphorylation and MAFbx-dependent degradation [105]. Together with RBP4-STRA6 signaling after denervation [49], these studies show why results from one wasting condition should not be used as a universal statement about filament selectivity.

Recent turnover work also clarifies what it does and does not measure. Deuterium-oxide labeling during recovery from disuse atrophy identified persistent proteostatic impairment and accumulation of insoluble ubiquitinated proteins in old muscle despite high myofibrillar synthesis [106]. A 2025 methodological review emphasized that deuterium-oxide-derived synthesis and turnover estimates depend on pool definition and interpretation [107]. These studies support contemporaneous measurements of synthesis, abundance, solubility, and degradation instead of reliance on a single fraction.

### 5.6. Minimum Reporting Needed to Interpret Sparing

Studies claiming selective sparing should report enough information to reconstruct the accessible pool. Reports should specify the buffer ionic strength, the detergents used, and each fraction analyzed. Recovery of a known insoluble thick filament marker should be shown. Reports should also provide muscle and fiber composition, neural and loading state, and time after the insult. Abundance should be normalized to total protein or starting tissue mass, and ubiquitination should be interpreted in the context of protein abundance. Tagged substrates must be shown to enter native filaments. When an E3 is assigned, binding, site-specific ubiquitination, loss of function, and rescue should be treated as separate evidentiary layers.

## 6. Biological Accessibility, Analytical Recovery, and Discriminating Experiments

### 6.1. Two Biological Determinants and One Analytical Layer

The reviewed evidence converges on two biological determinants and one observer-side analytical layer (Figure 1). The first biological determinant is assembly-dependent exposure. Polymerization, lattice packing, and associated proteins can influence encounters with E3 ligases, segregases, proteases, and antibodies. Ordered thick filament loss and delayed myofibril breakdown show that structural state matters [13,26,27,28]. The regional titin ubiquitination pattern is compatible with this idea, although its interpretation remains constrained by analytical coverage [29,43].

The second biological determinant is stability and damage-state regulation. Chaperones, kelch proteins, end-binding proteins, oxidation, and mechanical damage can change folding, assembly, or the probability of release. KLHL40-mediated preservation of LMOD3 and NEB and KLHL41-dependent nebulin stabilization exemplify direct stability control [16,17]. KLHL41-mediated NRAP destruction and BAG3-centered quality control show that the same systems can also promote removal of selected or damaged components [18,51,52,53]. Evidence for these mechanisms comes largely from developmental, myopathy, cardiac, or biochemical systems and should not be read as direct proof of their activity in adult atrophy.

The observer-side analytical layer is recovery and detection. Extraction, fraction selection, peptide detectability, blot transfer, and normalization determine which molecules enter a dataset. Low-salt under-recovery of MyHC, the special analytical difficulty of titin, and negative breakdown estimates caused by fraction redistribution show how recovery can mimic biological selectivity [15,43,50]. This analytical layer is not a third biological gate around the filament.

The two biological determinants and the analytical layer can coexist. A structurally protected protein may remain in an insoluble assembly and then be under-recovered by an assay. Conversely, a damaged protein may be released, modified, and removed from a myofibrillar fraction before degradation is complete. Figure 1 therefore separates specimen biology from observer-side analysis and represents proteasomal and autophagic routes as branches. Its lower panel summarizes possible processing steps and does not prescribe a required order. Recognition, ubiquitination, release, extraction, and route engagement may occur in a different sequence or may not all be required for every substrate.

### 6.2. The Framework Changes the Meaning of a Negative Result

Under this framework, a preserved band indicates the recovery pool was preserved, not intrinsic resistance. Failure to detect ubiquitination is evidence that the modified peptide was not detected under those conditions, not proof that no site was modified. The lack of genotype dependence shows that the tested ligase is not required for the net loss in that model, not that the ligase cannot recognize the protein. These distinctions are more than semantics. They determine which experiment logically follows.

Figure 2 maps the principal claims by evidence type without converting them into a composite grade. MuRF1-dependent thick filament loss has direct in vivo genetic and biochemical support, whereas relative thin filament sparing remains model-specific and is qualified by biochemical actin ubiquitination and analytical sensitivity. Model and extraction dependence have direct experimental support but limited clinical testing. KLHL40/KLHL41 stabilization has direct genetic and biochemical support in development or myopathy, not adult atrophy. MICAL1 regulates actin state in skeletal muscle cells, but an atrophy-specific oxidation-to-degradation pathway has not been demonstrated [64]. The central gray panel identifies a representative set of actin-binding proteins for which protein-specific half-life and degradation-route measurements during atrophy remain unavailable.

The evidence labels in Figure 2 apply to the claim and experimental context stated within each panel. Direct evidence means that the stated event was tested in that context through an in vivo genetic perturbation, a controlled intervention, or a biochemical demonstration of the proposed mechanism. Supportive evidence comes from a related system or assay that is mechanistically consistent but does not establish the same claim in adult skeletal muscle atrophy. Context-dependent evidence indicates that the observed pattern changes with the atrophy model, combined stimulus, extraction method, or measured fraction. Limited or not tested indicates that the relevant protein-specific half-life or degradation route has not been measured in the stated setting.

### 6.3. Discriminating Experiments

The most informative experiment would combine pulse-chase labeling with exhaustive fraction accounting. In the same muscle and time course, soluble, myofibrillar, detergent-insoluble, and total homogenate pools should be quantified for the same peptides. Protein abundance, synthesis, ubiquitin-site occupancy, and appearance in released fractions should be measured concurrently. High- and low-salt extraction could be performed on matched aliquots to estimate method-dependent recovery. This design would distinguish genuine destruction from release, assembly, and analytical loss.

A second experiment would stratify ubiquitination by assembly state. Candidate proteins could be expressed at near-endogenous levels and separated into monomeric, oligomeric, and filament-incorporated pools. E3 binding, site occupancy, chain topology, segregase recruitment, and degradation would then be compared across states. For actin, it is not sufficient to show that MuRF1 ubiquitinates purified protein. The critical test is whether the same sites and kinetics occur when actin is incorporated into a native thin filament.

A third priority is the access-setting proteins. CFL2, twinfilin, COTL1, drebrin, profilin, and selected capping proteins should undergo protein-specific half-life measurement across denervation, immobilization, fasting, glucocorticoid exposure, and cachexia, with neural and loading variables separated. Perturbing each regulator should be followed by actin polymerization state, filament incorporation, ubiquitin-remnant mapping, and muscle function. If regulatory-protein turnover precedes bulk actin loss and causally changes its accessibility, the access framework gains direct support.

A causal design should also separate recognition from access genetically. An inducible degron can acutely remove a candidate shield or end regulator after sarcomeres have matured, avoiding developmental confounding. In parallel, an E3-binding-defective but assembly competent substrate, an extraction-defective p97 perturbation, and a proteasome or autophagy blockade can locate the rate-limiting step. Epistasis can locate the relevant step. If removal of the shield exposes actin but p97 inhibition prevents loss, shielding acts upstream of extraction. If the substrate remains stable despite verified exposure and extraction, a downstream specificity determinant is required.

### 6.4. Falsification and Limitations

An access-based account is falsifiable. Its strongest form would be weakened if a native thin filament component were ubiquitinated with equal site occupancy and kinetics in a fully assembled myofibril and in solution, recruited segregases equally, yet remained selectively stable in vivo under validated quantitative extraction. Such a result would require a downstream determinant independent of access. Likewise, if paired whole-tissue and fraction-specific measurements reproduced a large selective loss under multiple extraction chemistries and across distinct atrophy models, analytical access would no longer be an adequate explanation for that phenotype.

The present synthesis has limitations. The apparent Cohen–Polge tension rests heavily on two landmark studies that used different systems [13,14]. MuRF1 also overlaps functionally with MuRF2 and MuRF3, which complicates inference from single-ligase perturbation [30,31,32,33]. The extraction argument is compelling, but it does not invalidate every report of preferential myosin loss [15,44]. Direct KLHL40 and KLHL41 studies are mainly developmental or myopathic [16,17,18]. The BAG3/CapZ evidence is largely cardiac [53,66], and the direct MICAL oxidation literature is primarily biochemical or non-muscle [58,59,60,61,62,63]. Skeletal muscle-cell evidence supports MICAL1-dependent actin remodeling but does not establish an atrophy pathway [64]. Human protein-turnover datasets remain small and physiologically heterogeneous [50,67,69]. The evidence base for the selected actin regulators remains incomplete and should be reassessed as new studies emerge.

## 7. Conclusions

The thin filament does not appear spared for one reason. In some models, specific ligases are not required for its net loss. In other contexts, actin is a competent ubiquitination substrate. Structural incorporation, stabilizing partners, damage state, extraction chemistry, and movement between fractions influence the observation obtained. Biological accessibility should not replace substrate specificity as an explanation. It should be evaluated alongside downstream determinants of fate and observer-side recovery. Claims of preferential degradation in intact muscle are strongest when the relevant biochemical forms, assembly states, fractions, and analytical recovery have been defined.

This ordering clarifies several long-standing discrepancies without declaring any single study incorrect. It also yields a concrete research program. Measure all fractions, map ubiquitination to assembly state, and determine the turnover of the actin regulators that open or close access to the filament. Until those measurements are made, a sparing phenotype should be treated as a conditional observation rather than a molecular identity. End-capping, oxidation, force-dependent damage, and segregase activity now provide specific candidate gates that can be placed experimentally upstream or downstream of ligase recognition.

## Figures and Tables

**Figure 1:**
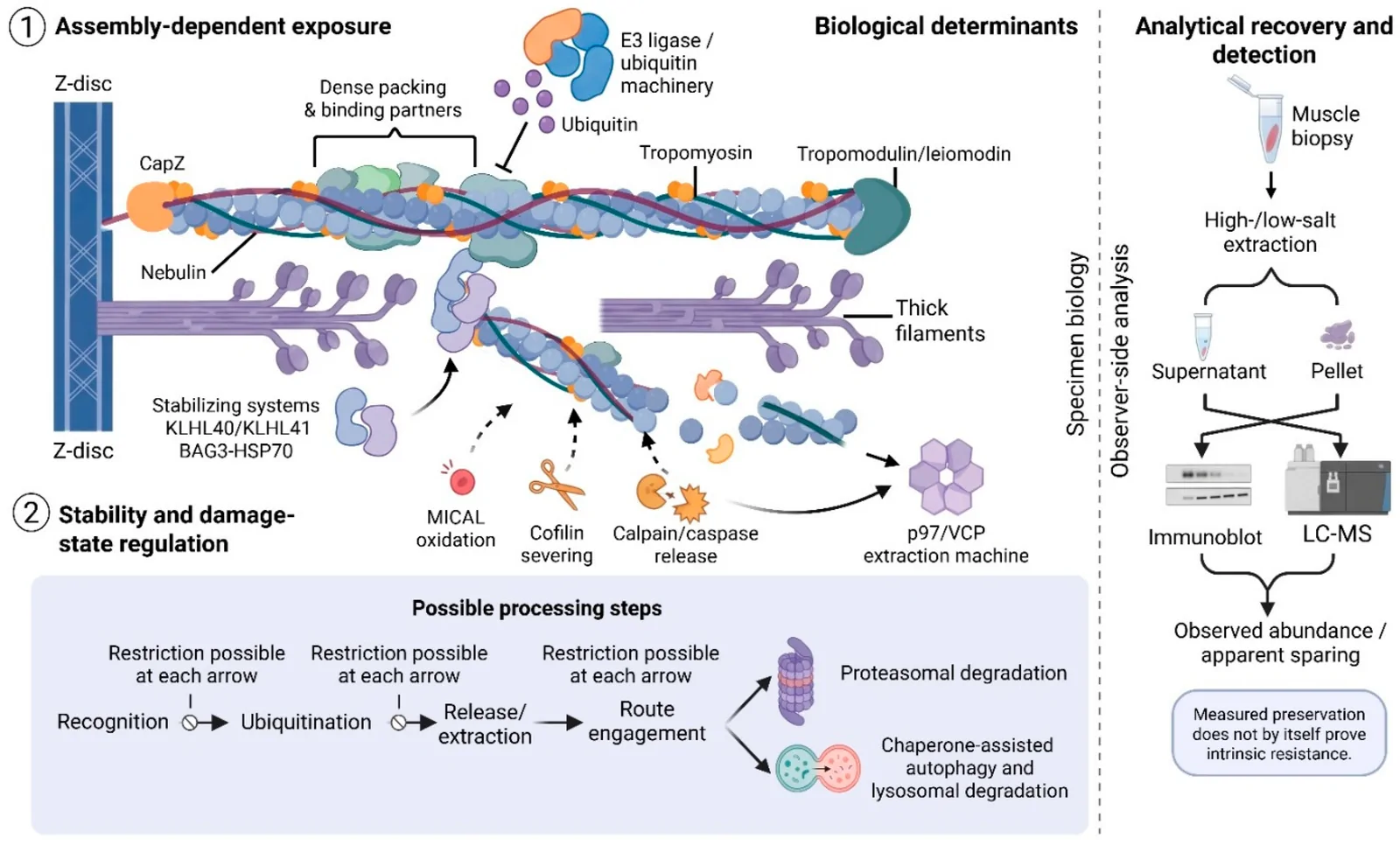
Two biological determinants and one observer-side analytical layer in the interpretation of myofibrillar protein loss. Assembly-dependent exposure and stability or damage-state regulation operate within the specimen. Analytical recovery and detection depend on extraction, fraction selection, blot transfer, peptide detectability, and normalization. Structural disruption, ubiquitination, p97/VCP-dependent extraction, proteasomal degradation, and chaperone-assisted autophagy are related but not obligatorily linear events. The lower panel summarizes possible processing steps whose order and requirement can vary by substrate and model. Restriction at one step can preserve the recovered pool without proving intrinsic resistance. Created in BioRender. Lee, W. (2026) https://BioRender.com/hodue8b (accessed on 22 September 2026).

**Figure 2:**
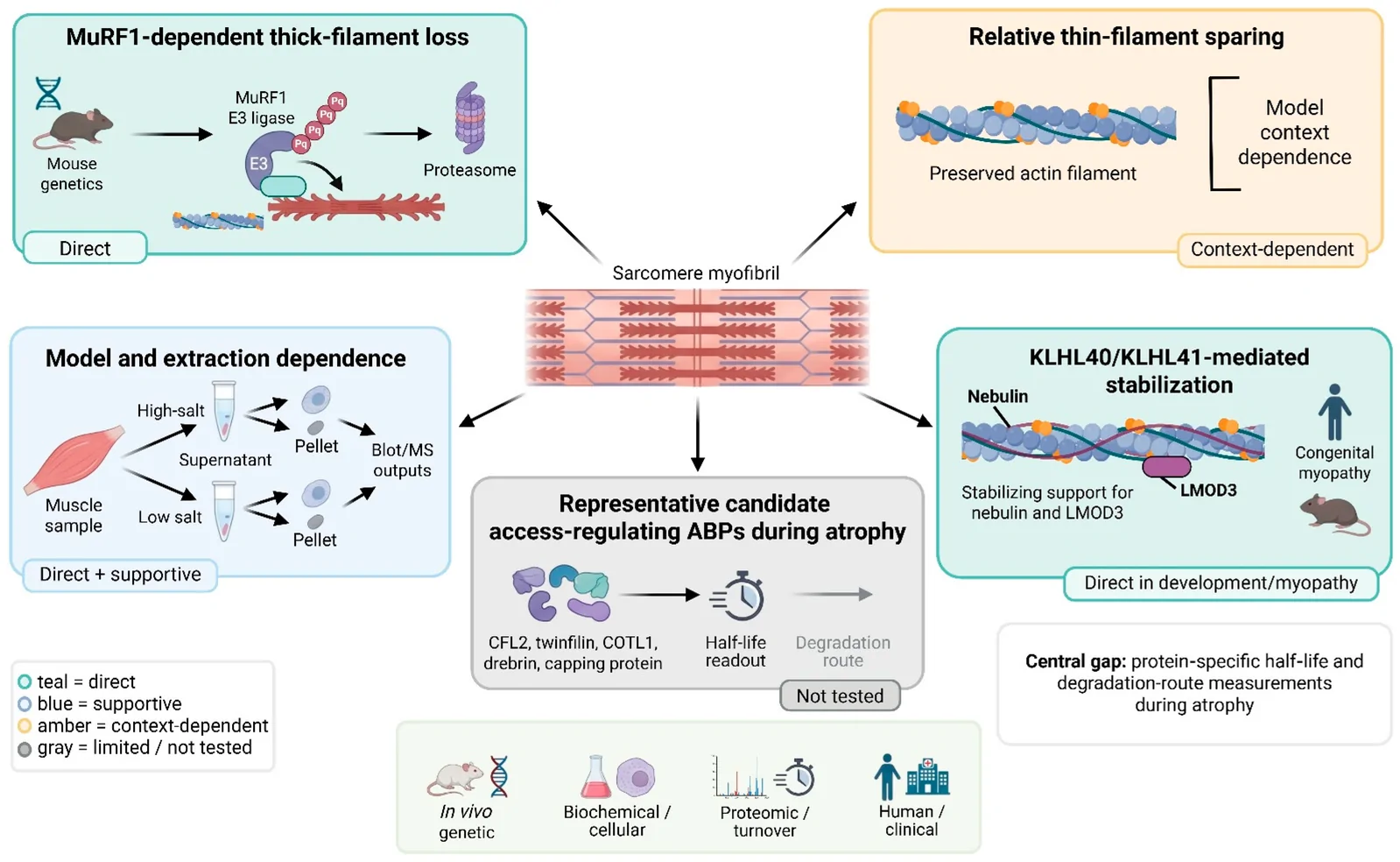
Evidence map for biological accessibility, stability regulation, and analytical recovery in muscle atrophy. Evidence labels are claim-specific. Direct denotes causal or mechanistic testing within the stated panel context. Supportive denotes related evidence from another system or assay. Context-dependent denotes variation across models or analytical conditions. Limited or not tested denotes an unmeasured protein-specific half-life or degradation route in the stated setting. Direct evidence in development or myopathy should not be generalized to adult atrophy. The central gray panel presents representative candidate access-regulating actin-binding proteins and does not define an exhaustive list. Primary studies are cited with the corresponding discussion in Section 2, Section 3, Section 4 and Section 5. Created in BioRender. Lee, W. (2026) https://BioRender.com/by6uph2 (accessed on 22 September 2026).

**Table 1:** Model and method dependence of reported myofibrillar protein selectivity.

Model or Stimulus	System	Analysis	Reported Pattern	Interpretive Boundary
Denervation	Mouse skeletal muscle	MuRF1 genetic perturbation; myofibrillar immunoblot	Ordered MuRF1-dependent thick filament loss; thin filament decline not MuRF1-dependent	Direct in vivo evidence for ligase requirement, not proof that actin cannot be ubiquitinated [13]
Fasting	Mouse skeletal muscle	MuRF1 genetic perturbation; myofibrillar immunoblot	MuRF1-dependent loss of selected thick filament components	Systemic catabolism without neuromuscular disconnection; substrate competence remains state-dependent [13]
Glucocorticoid	Myotubes and skeletal muscle	Immunoblot; cellular and biochemical ubiquitination	MuRF1-dependent MyHC degradation and actin ubiquitination competence	Tagged, soluble, or enriched pools do not establish tissue-level selectivity [14,36]
Cancer cachexia	Mouse skeletal muscle	Matched low- and high-salt extraction	Selective MyHC depletion not reproduced with adequate high-salt recovery	Low-salt supernatant under-recovers filamentous MyHC [15]
Cachexia plus denervation	Mouse skeletal muscle	Contractile-protein abundance under combined insults	Preferential myosin loss emerged with denervation but not cachexia alone	Neural and loading state modifies the systemic catabolic response [44]
Steroid plus denervation	Rat skeletal muscle	Protein abundance with or without electrical stimulation	Electrical stimulation attenuated preferential myosin loss	Contractile activity changes the observed phenotype [45]
Sepsis	Rat skeletal muscle	Myofilament release; calcium-dependent proteolysis	Release of actin- and myosin-containing material and calpain-sensitive proteolysis	Release can precede loss and does not identify an E3-substrate pair [39,40]

**Table 2:** What common measurements establish and what they do not.

Measurement	Positive Result Supports	Negative or Sparring Result Does Not Prove	Access Variable That Must Be Controlled
In vitro ubiquitination	Biochemical competence of an E3-substrate pair	Modification of the assembled native protein in vivo	Protein conformation, binding partners, polymerization state
Ligase loss-of-function with immunoblot	Ligase dependence of abundance in the recovered pool	Absence of ligase binding or ubiquitination in another pool	Extraction efficiency, fraction recovery, model and time
Diglycine-remnant proteomics	Presence and regulation of ubiquitinated lysine sites	Proteasomal destruction or complete substrate attribution to one E3	Protein abundance, peptide detectability, site occupancy, assembly state
Dynamic labeling of one fraction	Appearance or replacement within that fraction	Direct degradation when inter-fraction transfer is possible	Soluble-to-myofibrillar redistribution and abundance normalization
Preserved thin filament band	Stability of the assayed, extractable pool	Intrinsic resistance to every ubiquitin ligase	Insoluble material, masked epitopes, filament packing

## Data Availability

No new data were created in this study. All data discussed are available in the cited primary publications.
